# Impacts of water hardness and road deicing salt on zooplankton survival and reproduction

**DOI:** 10.1038/s41598-023-30116-x

**Published:** 2023-02-20

**Authors:** Aniyah Woodley, Leslie L. Hintz, Bayley Wilmoth, William D. Hintz

**Affiliations:** Department of Environmental Sciences and Lake Erie Center, The University of Toledo, 6200 Bay Shore Road, Oregon, OH 43616 USA

**Keywords:** Freshwater ecology, Urban ecology

## Abstract

Rising salinity from road deicing salts threatens the survival and reproduction of freshwater organisms. We conducted two experiments to address how *Daphnia pulex* survival and reproduction were affected by road salt concentration (control, 120, 640 and 1200 mg Cl^−^/L) crossed with three concentrations of water hardness (20, 97, 185 mg CaCO_3_ /L). *D. pulex* survival was poor in our hard water treatment in both experiments (185 mg CaCO_3_ /L), potentially indicating a low tolerance to hard water for the strain used in our experiments. With the remaining two hardness treatments (20 and 97 mg CaCO_3_ /L), we found no evidence of an interactive effect between salt concentration and water hardness on *D. pulex* survival. In our population-level experiment, *D. pulex* survival was reduced by > 60% at 120 mg Cl^−^/L compared to the control. In the individual experiment, survival was similar between the control and 120 mg Cl^−^/L, but ≤ 40% of individuals survived in 640 and 1200 mg Cl^−^/L. For the surviving individuals across all treatments, the number of offspring produced per individual declined with increasing Cl^−^ concentration and in hard water. Our results indicate that current Cl^−^ thresholds may not protect some zooplankton and reduced food availability per capita may enhance the negative impacts of road salt.

## Introduction

Human-induced salinization caused by the application of road deicing salts reduces the survival, growth, and reproduction of freshwater organisms^1–4^. After application, road salts wash into surface and ground waters during spring snowmelt or precipitation events leading to substantial increases in the salinity of freshwater lakes across cold regions worldwide^5–8^. Chloride (Cl^−^) concentration is often a signal of road salt pollution because many of the most common deicing salts are the inorganic salts sodium chloride (NaCl), magnesium chloride (MgCl_2_), and calcium chloride (CaCl_2_). Among these salt types, NaCl is the most frequently used deicer because it is most economically feasible compared to the other salts and alternative deicers^9^.

Road deicing salts have been applied for more than 80 years. They are applied in North and South America, Europe, and Asia in regions that experience snow and ice during the winter^9^. The ecological impacts of these salts have become more clear in recent decades^4,10,11^. Elevated road salt concentrations in lakes have negative impacts on species across trophic levels, community interactions, and ecosystem processes^3,4^. Multiple studies show road salts trigger declines in survival, growth, reproduction, population size, and diversity of freshwater organisms^12–16^. In some lakes, these ecological impacts can occur between 4 and 40 mg Cl^−^/L, well below the lowest Cl^−^ thresholds designed to protect freshwater organisms, which is 120 mg Cl^−^/L in Canada^17^.

As the ecological impacts continue to emerge in the scientific literature, several studies indicate that water hardness can play a major role in the toxicity of salts used as deicers^17–20^. Results from these studies suggest that road salts may become more toxic as water hardness decreases. It is unclear from these studies if a lower limit threshold exists at which the influence of road salt toxicity increases. Freshwater lakes, in particular, vary widely in water hardness^21^, which is often measured as the concentration of calcium carbonate (CaCO_3_). Fresh waters are generally considered “soft” between 0 and 60 mg CaCO_3_/L, “moderately hard” between 61 and 120 mg CaCO_3_/L, “hard” between 121 and180 mg CaCO_3_/L, and “very hard” when > 180 mg CaCO_3_/L^22^. Towards developing thresholds for Cl^−^ that protect freshwater organisms, it is essential to understand the interactive nature between road salt toxicity and water hardness.

Food availability and competition can potentially affect road salt toxicity^23,24^. For instance, the 14-day lethal concentration at which only 50% of a population survives (i.e. an LC_50_) goes up from about 50 mg Cl^−^/L to > 250 mg Cl^−^/L when food concentration increases from 0.2 to 1.0 mg carbon/L^23^. Many freshwater lakes vary in their trophic status where some waters have considerably lower food availability per capita than more productive systems. Thus, understanding how food availability per capita affects the survival of freshwater organisms could enhance how we manage salinization for lakes of different trophic status.

Our goal was to determine if water hardness and road salt concentration interact to affect the survival and reproduction of *Daphnia pulex*, a ubiquitous zooplankton species commonly found in lakes and many wetlands. We conducted two separate experiments to address our goal. First, we conducted a population-level toxicity test to determine the interactive effects of road salt concentration and water hardness on *D. pulex* survival. Second, we conducted an individual-level experiment to determine if road salt concentration and water hardness had interactive effects on the survival, timing of first reproduction, and brood size of *D. pulex*. We hypothesized that higher road salt concentrations would decrease the survival of *D. pulex* in the population-level experiment, with a larger negative effect size occurring in soft water compared to moderately hard and hard water. In the individual-level experiment, we hypothesized that soft water combined with higher salt concentrations would yield lower survival, smaller brood size, and increased time to first brood when compared to soft water with no salt and salt-laden water with higher water hardness. We also expected that a lower food availability per capita would reduce survival in the population experiment at the chosen Cl^−^ concentrations compared to the individual-level experiment.

## Results

### Population survival experiment

In the hard water treatment, only one individual survived to the end of the experiment. Thus, we were unable to include the hard water treatment in the statistical analyses for the population-level experiment, which resulted in two water hardness treatment levels instead of three in our statistical analyses.

**Figure 1:**
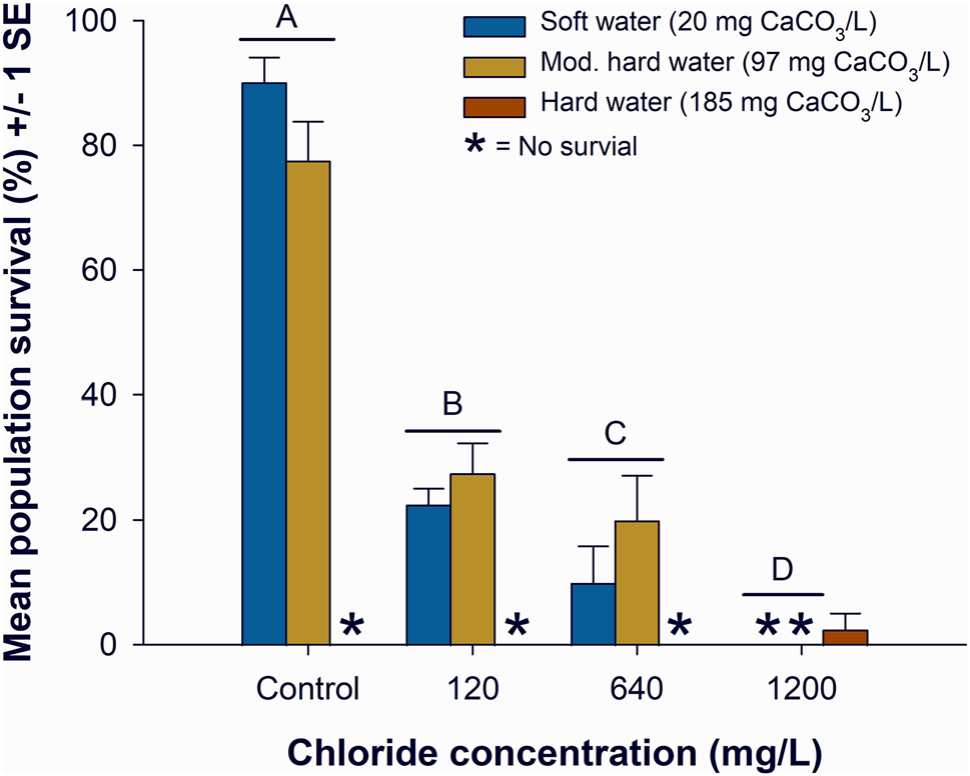
Mean percent survival of *Daphnia pulex* ± 1 standard error (SE) in the control and three road salt concentrations after an 11-d exposure. Different letters indicate main-effect differences in survival between chloride treatments. Note that the full suite of treatment combinations are shown here and main effects for chloride are indicated by different letters where *P* < 0.050 for a Holm-Sidak post-hoc test.

With the remaining two hardness treatment levels (20 and 97 mg CaCO_3_/L), we tested for an interaction between water hardness and road salt concentration with two-way ANOVA. The two-way ANOVA met the assumptions of normality (Shapiro–Wilk *P* = 0.096) and equal variance (Brown-Forsythe *P* = 0.103). We found no interaction between water hardness and road salt concentration in the two-way ANOVA (F_3,24_ = 2.21, *P* = 0.113). There was also no statistical evidence to suggest that there was a main effect of soft and moderately hard water on *D. pulex* (F_1,24_ = 0.04, *P* = 0.849). We found an effect of salt concentration on *D. pulex* survival (F_3,24_ = 127.54, *P* < 0.001; Fig. 1). As salt concentration increased survival decreased and all salt treatments were statistically different from each other. Average survival was 84% (1 SE =  ± 4.2%) in the control, 25% (1 SE =  ± 2.7%) in 120 mg Cl^−^/L, 15% (1 SE =  ± 4.6%) in 640 mg Cl^−^/L, and no *D. pulex* survived in 1200 mg Cl^−^/L (all *t* ≥ 2.2, all *P* ≤ 0.040).

### Individual survival and reproduction experiment

**Figure 2:**
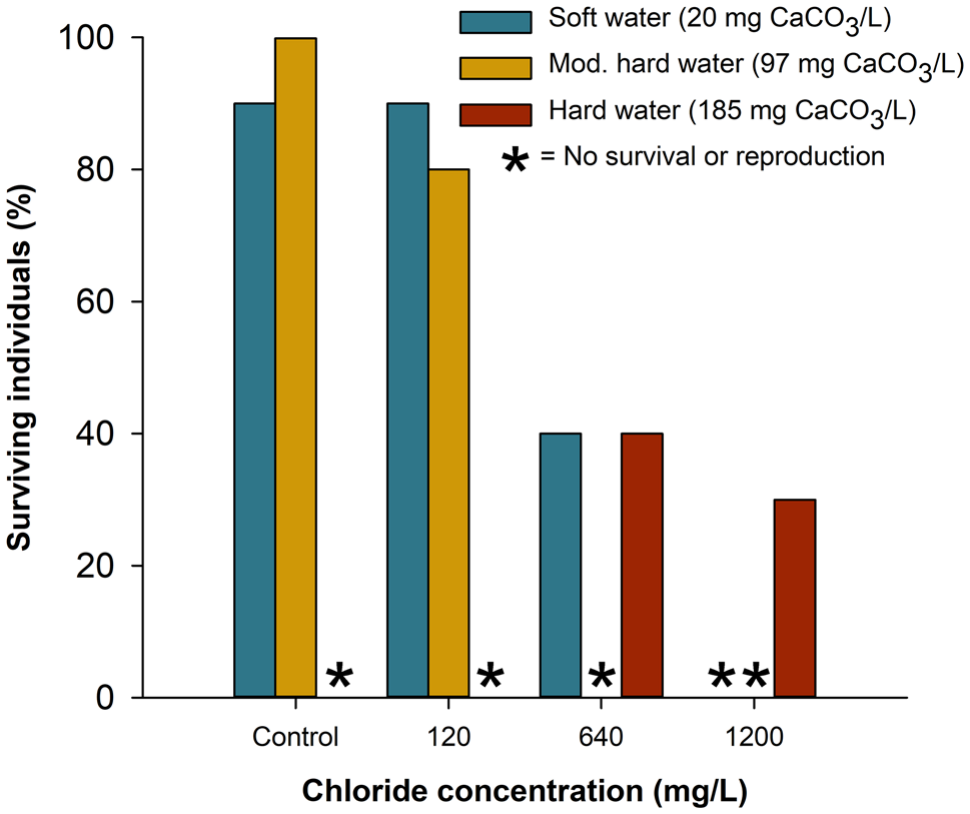
Percent survival of individual *Daphnia pulex* among three elevated road salt and water hardness concentrations. Data show the full suite of experimental treatments because no survival occurred in hard water in the two lowest chloride concentrations, but there was no indication of an interaction between water hardness and road salt concentration on survival in the remaining lower water hardness treatments. Each treatment combination was replicated with 10 individual *D. pulex*, which is why no variance in survival is illustrated.

Survival of *D. pulex* varied among the experimental salt treatments (Fig. 2). In the soft water treatment (20 mg CaCO_3_/L), survival was 90% in the control and the 120 mg Cl^−^/L salt treatment. As salt concentration increased to 640 mg Cl^−^/L, survival decreased to 40% and none of the *D. pulex* survived in the highest salt treatment of 1200 mg Cl^−^/L. In the moderately hard water treatment (97 mg CaCO_3_/L), survival was 100% in the control and 80% in 120 mg Cl^−^/L. No *D. pulex* survived in the 640 and 1200 mg Cl^−^/L treatments. In the hard water treatment (185 mg CaCO_3_/L), none of the *D. pulex* survived in the control or the 120 mg Cl^−^/L salt treatment. Surprisingly, survival was 40% and 30% in the 640 and 1200 mg Cl^−^/L treatments, respectively.

**Figure 3:**
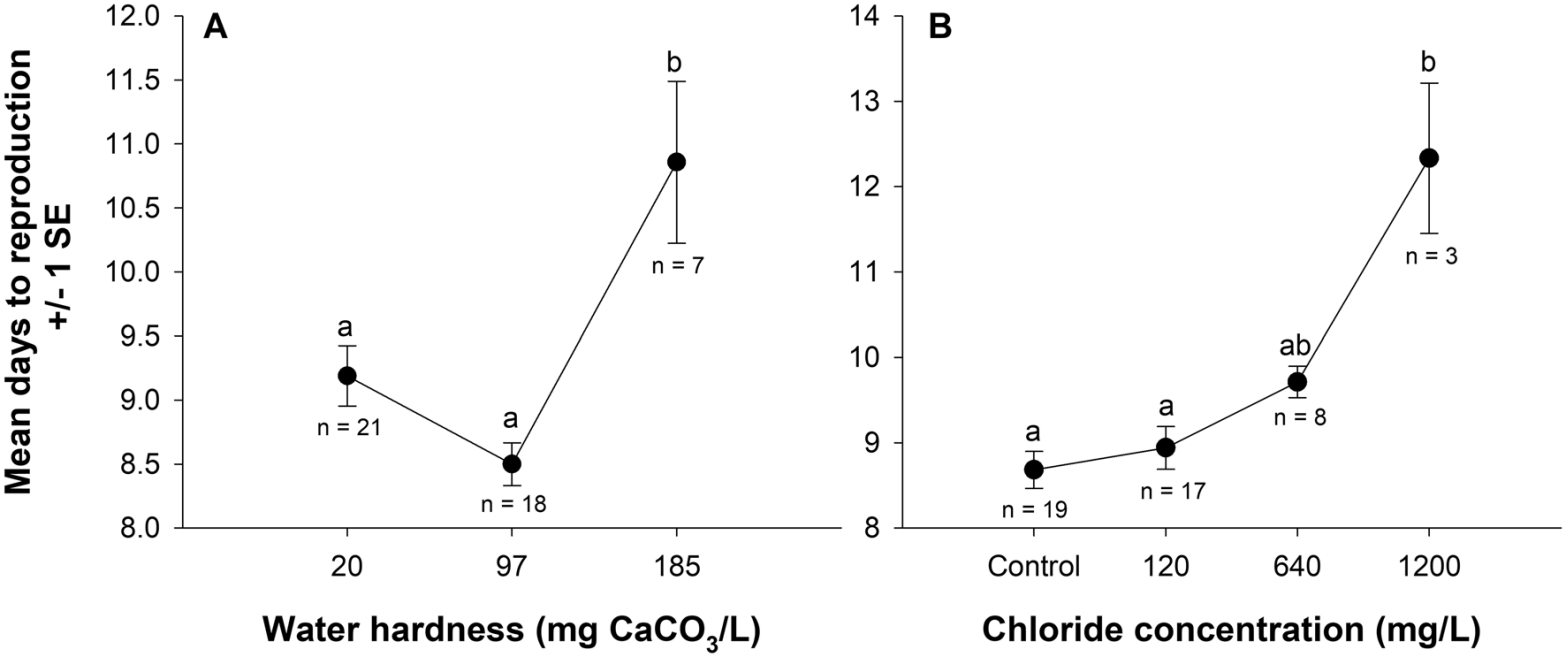
Effects of water hardness (**A**) and chloride concentration (**B**) on the average number of days ± 1 standard error (SE) to first reproduction in *Daphnia pulex*. We found no evidence of an interaction between water hardness and road salt concentration. Letters indicate statistical differences from an independent samples Kruskal–Wallis test with Bonferroni-corrected pairwise comparisons where *P* ≤ 0.058 for water hardness and *P* ≤ 0.027 for chloride concentration. Sample sizes are indicated below the response for each treatment.

Among the surviving individuals, we found no interaction between water hardness and salt concentration on *D. pulex* time to reproduction (Wald *Χ*^2^ = 0.001, *P* = 0.996). We did find a main effect of water hardness on time to reproduction (Kruskal–Wallis H = 14.2, df = 2, *P* < 0.001). Time to reproduction was 19–28% longer (1.7–2.4 d, *P* ≤ 0.058) in hard water compared to soft and moderately hard water (Fig. 3A). We also found an effect of salt concentration on time to reproduction (Kruskal–Wallis H = 14.6, df = 3, *P* = 0.002). Time to reproduction was similar between the control, 120 mg Cl^−^/L, and 640 mg Cl^−^/L. Time to reproduction was 43% longer in 1200 mg Cl^−^/L (3.7 d, *P* = 0.007) compared to the control (Fig. 3B).

**Figure 4:**
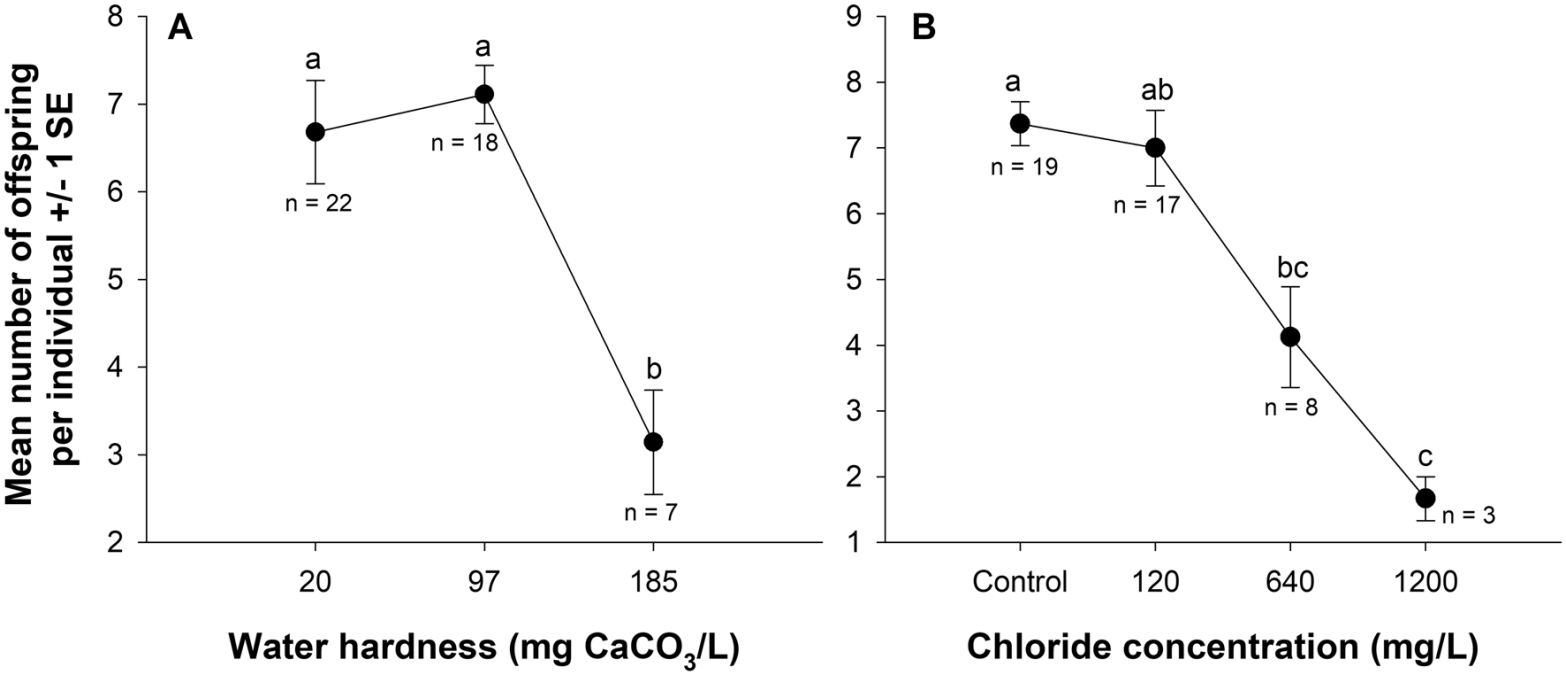
Effects of water hardness (**A**) and chloride concentration (**B**) on the average number of offspring produced ± 1 standard error (SE) in *Daphnia pulex*. We found no interaction between water hardness and road salt concentration. Letters indicate statistical differences from an independent samples Kruskal–Wallis test with Bonferroni-corrected pairwise comparisons where *P* ≤ 0.005 for water hardness and *P* ≤ 0.041 for chloride concentration. Sample sizes are indicated below the response for each treatment.

**Figure 5:**
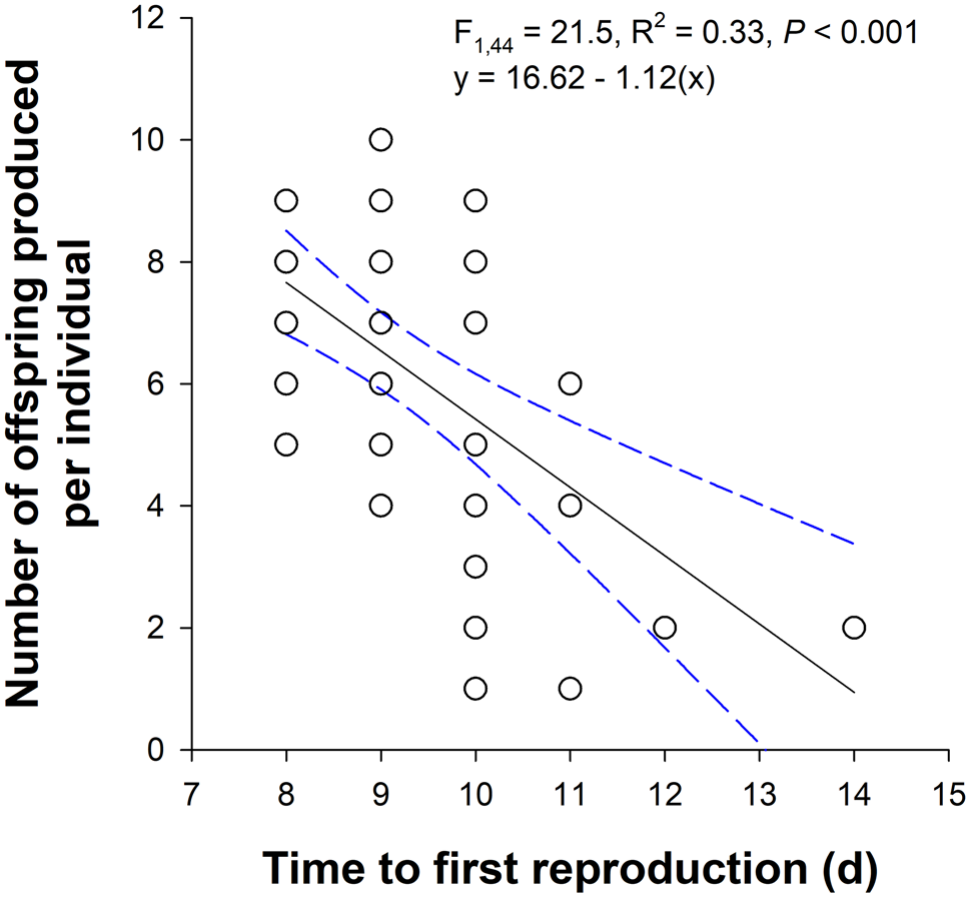
Negative relationship between the day *Daphnia pulex* first reproduced and the number of offspring produced. Solid black line represents the regression line and blue, dashed lines indicate the 95% confidence intervals (note: some data points overlap).

We found no interaction between water hardness and salt concentration on the number of neonates produced among the surviving individuals (Wald *Χ*^2^ = 0.42, *P* = 0.515). We did find a main effect of water hardness (Kruskal–Wallis H = 11.8, df = 2, *P* = 0.003) and road salt concentration (Kruskal–Wallis H = 15.3, df = 3, *P* = 0.002) on the number of offspring produced per individual (Fig. 4). In hard water, offspring production was 54% lower (*P* = 0.003) and 56% lower (*P* = 0.005) than the soft and moderately hard water treatments, respectively. Offspring production was reduced by 45% in 640 mg Cl^−^/L compared to the control (*P* = 0.028). We found weak statistical evidence that 41% fewer offspring were produced in the 640 mg Cl^−^/L compared to the 120 mg Cl^−^/L (*P* = 0.077). Compared to the control and 120 mg Cl^−^/L, offspring production was reduced by 78% in 1200 mg Cl^−^/L (*P* ≤ 0.041). Lastly, we found an inverse relationship between day of first reproduction and the number of offspring produced per individual—individuals where delayed reproduction occurred produced fewer neonates (Fig. 5).

## Discussion

We tested how water hardness influences the effects of NaCl road salt because many lakes vary widely in water hardness and road salt has been shown to have very different effects along a water hardness gradient. We are limited to discussing our results primarily based on the low and moderately hard water treatments due to high mortality of the controls in the high water hardness treatment in both experiments. Among these two water hardness treatments, previous research indicates that soft water will trigger a greater negative response by freshwater organisms to road salts^17,18,20,25^. Soft water ranges from 0 to 60 mg CaCO_3_/L^22^. Our soft-water treatment was 20 mg CaCO_3_/L, falling on the lower half of the soft water spectrum. Yet, we found no interaction between the two water hardness concentrations and road salt concentration in the population experiment or the individual experiment. Our results did show that elevated road salt concentration reduced the survival of *D. pulex* by > 60% in the population experiment at the Canadian chronic threshold of 120 mg Cl^−^/L regardless of soft or moderately hard water. In the individual experiment, survival and reproduction were similar between the control and 120 mg Cl^−^/L, but were reduced in the acute threshold of 640 mg Cl^−^/L. Our results indicate that while road salt did not interact with the two lower water hardness treatments, the Cl^−^ thresholds tested in our experiment might not be low enough for some zooplankton under similar conditions tested here.

The effects of NaCl road salt in our experiment yielded different results in a population- versus individual-level context. Different impacts of road salts on populations versus isolated individuals have occurred in other salinization studies^12^, but also in response to other contaminants such as Cu and Pb^26^. Food availability per capita was lower in our population experiment compared to our individual experiment and the decline in survival that occurred in 120 mg Cl^−^/L in the population experiment but not the individual experiment could have been due to reduced food availability^23,27^. Brown and Yan^23^ illustrate that most toxicological bioassays have an over-abundance of food so that food availability is not a limiting factor or additional stressor on the organism being examined, which is not always the case in natural environments. We cannot know if survival would have been different if we had a high and low food treatment for both experiments, but such a design would better elucidate the mechanisms behind food availability and road salt toxicity. Further, with an increasing number of conspecifics, competition for space can be intense in *D. pulex*^24^. In both experiments, survival was on average between 80 and 100% in the controls, indicating that space probably was not limiting in the population experiment, which supports food availability per capita as the possible mechanism for lower survival in 120 mg Cl^−^/L in the population experiment. Ultimately, sound water quality guidelines will need to consider resource restrictions in natural populations that may trigger different responses to contaminants than single-individual experiments with abundant food.

The negative effect of the road salt on survival and offspring production in *D. pulex* suggests that current guidelines for chronic and acute exposure of Cl^−^ in freshwater organisms may not be low enough to protect some zooplankton and other sensitive organisms in some environmental contexts. The Canadian chronic threshold is one of the lowest known Cl^−^ thresholds globally set by a federal government to protect freshwater organisms^28–31^. Multiple studies show that current water quality guidelines for Cl^−^ are not protective of several zooplankton taxa and substantial reductions in abundance can occur at established Cl^−^ thresholds^17,32,33^. Recent evidence suggests that some zooplankton species experience reductions in survival and reproduction well below 120 mg Cl^−^/L. For instance, Valleau et al.^34^ found that the 14-d LC_50_ for two freshwater zooplankton, *Bosmina longirostris* and *Chydorus brevilabris*, from soft water lakes ([Ca^2+^] = 1.3–1.4 mg/L, 1.7–3.5 mg CaCO_3_/L) were 24 mg Cl^−^/L and 60 mg Cl^−^/L, respectively. Arnott et al.^17^ found that increased mortality and reduced reproduction can occur at 4–40 mg Cl^−^/L in Canadian Shield lakes, perhaps due to very low concentrations of Ca^2+^ and other ions like Mg^2+^. While we did not find any interaction between road salt and water hardness, our evidence supports lowering Cl^−^ thresholds to protect freshwater zooplankton and limnetic food webs.

It is important to consider our treatment concentrations when discussing the interaction between water hardness and road salt concentration. We chose environmentally relevant concentration of water hardness observed among ecosystems affected by road salt. It is possible that categorical designations of water hardness may not reflect a concentration range where we should expect road salt to have an increased, reduced, or no effect on zooplankton. To illustrate, as previously discussed, our soft-water treatment was on the lower end of the water hardness scale^21,22^. However, our soft-water treatment was perhaps not low enough to detect an interaction with road salt. Characteristics of the soft-water FLAMES medium used in studies where the impacts of road salt occur at very low Cl^−^ concentrations—such as Valleau et al.^34^—has 9.41 mg CaCO_3_/L, 2.54 mg Ca^2+^/L, and 0.75 mg Mg^2+^/L^35^. These concentrations are lower than our soft-water treatment of 20 mg CaCO_3_/L. It is possible that our soft water was not “soft enough” in the soft water hardness category to elicit an interactive response with our road salt concentrations. It is also possible a threshold exists within the soft-water category whereby road salts have a greater negative impact. Such a threshold would be important to identify if new chloride guidelines are established.

While we are unable to identify a water hardness concentration that increased or decreased the response of *D. pulex* to road salt, other studies have found evidence and made recommendations for modifying water quality guidelines related to Cl^−^ for different species. Elphick et al.^18^ suggested that Cl^− ^thresholds in waters with 10 mg CaCO_3_/L should be reduced to 64 mg Cl^−^/L. For our soft-water treatment of 20 mg CaCO_3_/L, Elphick et al.^18^ suggests the water quality objective for Cl^−^ should be 145 mg Cl^−^/L. Our results on the reproductive responses of *D. pulex* would generally support this water quality objective. However, population survival was reduced at 120 mg Cl^−^/L regardless of water hardness, perhaps reinforcing the idea that food availability in a population matters when considering Cl^−^ targets. Further research on how food availability, ecological community interactions, and water chemistry influence the impacts of road salts is certainly needed to produce the context for protective Cl^−^ thresholds.

Strangely, *D. pulex* survival in the hard water treatment was highest in the highest Cl^−^ concentrations and there was no survival in hard water in the control and low Cl^−^ concentrations. We had good survival in our controls in our soft and moderately hard water treatments, suggesting something unique occurred in the hard water treatment. It is recommended that *D. pulex* be cultured in moderately hard water and *D. pulex* can also persist in soft water as well^36,37^. *D. pulex* has also been used in experiments with hard-water treatments^38^. Thus, the survival of *D. pulex* in hard water in only the two highest Cl^−^ concentrations is perplexing. It could be possible that the salts in the EPA water recipe interacted with the NaCl road salt at a high concentration that somehow facilitated survival, but we are unaware of the mechanisms behind such an interaction. Whatever the mechanism, it is clear that the strain of *D. pulex* used in our experiment was not tolerant of our hard water treatment. Future work could explore how different strains of ubiquitous species like *D. pulex* vary in their responses to contaminants like road salts in lakes of varying water hardness.

### Conclusions

Our results show that current Cl^−^ thresholds may need to be reassessed and natural interactions such as competition and food availability need to be considered in such a reassessment. Further, we must consider a broader context for the interaction between road salt and water hardness that includes the freshwater salinization syndrome^7,8,39,40^. Protective Cl^−^ thresholds will need to account for the increasing multitude of co-occurring salts and ions in fresh waters that might interact additively, synergistically, or antagonistically with road salt pollution. Other stressors such as changing water temperatures due to climate change, pesticides, and nutrient pollution also have the potential to interact with road salts^41–43^. Ultimately, we will need to enhance our understanding of these numerous, potentially interacting and co-occurring stressors to fully understand the threat road salt salinization will pose to freshwater organisms.

## Methods

### Study organism and culturing

We conducted our study at the Experimental Freshwater Ecology Laboratory at The University of Toledo’s Lake Erie Center (Ohio, USA). We cultured* D. pulex* in the laboratory for nine months prior to the initiation of this study (strain from Aquatic Biosystems, Fort Collins, CO, USA). We used dechlorinated tap water from the City of Oregon, OH (USA) and diluted it with 50% reverse osmosis (RO) water. Lake Erie is the source of the tap water. It was diluted with RO water to ensure limited exposure of the *D. pulex* to chloride ions during the culturing phase. The Cl^−^ concentration of the culture medium ranged from 10 to 18 mg Cl^−^/L and water hardness as CaCO_3_ was approximately 35–45 mg/L, which is within the higher range of soft water^22^. Cultures were maintained in 15 L plastic containers filled with approximately 5 L of culture medium. Organisms were reared at 20 ± 2 °C under a 16 h light: 8 h dark photoperiod and were fed ad libitum three times per week with concentrated *Raphidocelis subcapitata*.

### Test media

To examine the influence of water hardness on road salt toxicity in *D. pulex* we conducted a population survival experiment and an individual survival and reproduction experiment. Both experiments used a factorial design that crossed four concentrations of Cl^−^ with three water hardness concentrations for a total of twelve treatments. The Cl^−^ concentrations (7, 120, 640 and 1200 mg Cl^−^/L) and water hardness concentrations (20, 97, 185 mg CaCO_3_/L) used in our experiments are environmentally relevant as they are within the ranges of concentrations found in freshwater ecosystems across North America. The concentrations of 120 and 640 mg Cl^−^/L were specifically chosen because they are the current Canadian guidelines for chronic and acute exposure to Cl^−^ in freshwater ecosystems and are the lowest known federal guidelines to protect freshwater organisms.

For both experiments, reconstituted water was prepared at each of the water hardness concentrations. This was done by adding reagent grade salts to aged tap water augmented with RO water (20% dechlorinated tap water: 80% RO water). The masses of the reagent grade salts added were manipulated to attain the appropriate hardness by taking into account the approximate hardness of the diluted tap water and referencing United States Environmental Protection Agency (EPA) protocols for the preparation of reconstituted water^36^. A 240 ml sample of each reconstituted water type was taken for later confirmation of the hardness concentration. We analyzed hardness using Method 10293 on a HACH DR3900 laboratory spectrophotometer (HACH, Loveland, CO, USA).

Aliquots of the three types of reconstituted water were dosed with halite (SafeStep® Ice Melter Sodium Chloride 3300, Overland Park, KS, USA) to achieve the elevated Cl^−^ concentrations (120, 640 and 1200 mg Cl^−^/L). While we did not use laboratory-grade NaCl, the use of actual rock salt for deicing has the advantage of attributing the impact to a commonly used deicer and any potential impurities rather than laboratory-grade NaCl, which may not reflect the full suite of potential ecological impacts. The 7 mg Cl^−^/L treatments served as the controls as this was the average concentration of the reconstituted water prior to any road salt addition. Chloride concentrations of the treatments were measured using a YSI ProDSS Multiparameter Water Quality Meter (YSI, Yellow Springs, OH, USA) and were confirmed to be within 10% of the nominal concentrations for all experimental treatments.

### Population survival experiment

**Figure 6:**
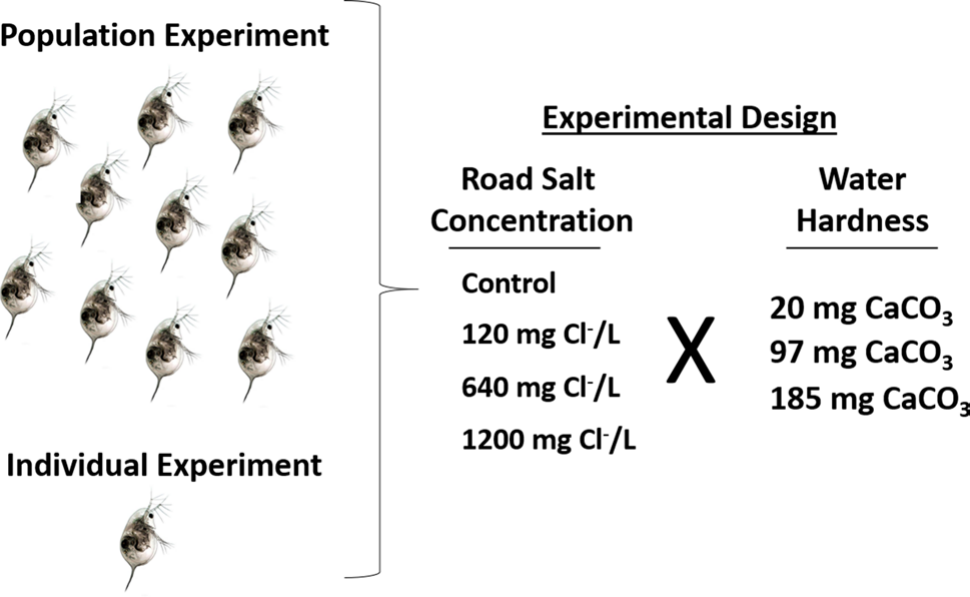
Experimental design of the population- and individual-level experiments to evaluate the potential interactive effects between road salt concentration and water hardness on zooplankton survival and reproduction.

In the survival experiment, we exposed *D. pulex* to four Cl^−^ concentrations (7 [control], 120, 640 and 1200 mg Cl^−^/L) at three different water hardness concentrations (20, 97, 185 mg CaCO_3_ /L) for 11 d (Fig. 6). We chose this time period because we did not want the population size to reach carrying capacity and start cycling since *D. pulex* can reproduce every few days^44^. There were four replicates of each treatment. For each replicate, 10 *D. pulex* neonates < 24-h old were placed in a beaker containing 500 ml of appropriately dosed test medium. The experiment was carried out in an environmental chamber at 21 (± 1)°C under a 16 h light: 8 h dark photoperiod. The beakers were lightly covered to reduce evaporation and maintain chloride concentration throughout the experiment. We fed each beaker with concentrated *Raphidocelis subcapitata* at a concentration of 25,000 cells/mL on Day 2, 4, 7 and 9 of the experiment, which was less that the individual experiment to reduce the food availability per capita. By Day 11 the neonates had developed into adults and we recorded the number of surviving individual adults. Individuals were classified as dead if no movement was observed after gentle prodding with a plastic transfer pipette. Another reason we stopped the experiment at this time point was because the adults began producing neonates and it would have been too difficult to track reproduction by individuals in a population.

### Individual survival and reproduction experiment

To evaluate the effects of road salt and water hardness on survival and reproduction of individual *D. pulex*, we exposed individuals to four Cl^−^ concentrations (7 [control], 120, 640 and 1200 mg Cl^−^/L) at three different water hardness concentrations (20, 97, 185 mg CaCO_3_ /L) until first reproduction occurred (Fig. 6). We placed *D. pulex* neonates (< 24-h old) into plastic cups (1 individual/cup) containing 70 mL of appropriately dosed test media. Each treatment was replicated 10 times. This experiment was conducted in the laboratory where temperature was maintained at 22 ± 1 °C and the photoperiod was set to a 16 h light: 8 h dark cycle. Individuals were fed 20,000 cells/mL of concentrated *Raphidocelis subcapitata* daily. We changed the water twice during the experiment by gently transferring each individual using a plastic transfer pipette to a new cup with test media. We monitored the experiment daily and survival, age at first reproduction, and size of first brood were recorded. Once an individual produced its first brood, we removed it from the experiment and recorded the time it took to produce the brood and the brood size (number of neonates).

### Data analysis

For the population survival experiment, we analyzed the effects of water hardness and salt concentration and their interactions on % survival (n = 4/treatment) using a two-way analysis of variance (ANOVA) because the design was balanced. A Dunn-Sidak post hoc test was conducted to examine pairwise comparisons if statistical support indicated an interactive or main effect of the experimental treatments. The parametric assumptions of normality were checked with a Shapiro–Wilk test and equal variance was checked with a Brown-Forsythe test, and the two-way ANOVA met these assumptions.

We used generalized linear models (GLM) to analyze the potential interactive effect of water hardness and salt concentration on the time to first reproduction and the number of offspring produced of surviving *D. pulex* in the individual experiment. One individual that survived to reproduction was removed from the analysis because this outlier took 22 d to reproduce while all other individuals reproduced within the interval of 8–14 d. The GLMs consisted of a different probability distributions and link functions, and the best model was chosen after assessing the Akaike’s Information Criterion (AIC) to determine the best fit model. An omnibus test revealed all fitted models were better than the intercept-only models. During data exploration, however, we found that the data for pairwise comparisons were non-parametric and did not meet the assumptions of normality and equal variance. In the absence of evidence for an interaction between water hardness and salt concentration, we used an independent-samples Kruskal–Wallis test to determine the main effects of water hardness and salt concentration on time to first reproduction and average number of offspring (i.e. neonates) produced per surviving and reproducing individual. Pairwise comparisons as part of the Kruskal–Wallis test examined whether there were differences among the different levels of hardness and salt concentration. The *P*-values in the pairwise comparisons were Bonferroni-corrected to account for the multiple comparisons. We chose this analysis because of the highly unbalanced sample size due to variation in survival among the experimental treatments, which can constrain many statistical procedures. We conducted these analyses in IBM SPSS Statistics 28.

## Data Availability

Data available on request from the corresponding author.
